# Impact of the coronavirus (COVID-19) disease pandemic on healthy lifestyle behaviors in persons with and without disabilities in Qatar

**DOI:** 10.3389/fpsyt.2023.1279663

**Published:** 2024-01-08

**Authors:** Uma Pandiyan, Brijesh Sathian, Ibin Kariyathankavil, Rafat Saad, Fatma Al Kuwari, Poovathoor Joseph Alexander, Saquib Hanif, Loubna Zabat

**Affiliations:** ^1^Qatar Rehabilitation Institute, Hamad Medical Corporation, Doha, Qatar; ^2^Geriatrics and Long-Term Care Department, Rumailah Hospital, Hamad Medical Corporation, Doha, Qatar

**Keywords:** COVID-19, people with disabilities, healthy lifestyles, lack of physical activity, physical health, mental health, Qatar

## Abstract

**Purpose:**

This study aimed to examine the impact of the COVID-19 pandemic on the lifestyle behaviors which included physical activity, sedentariness, healthy eating habits, sleep habits, and tobacco use in persons with and without disabilities in Qatar.

**Methods:**

This cross-sectional study used a structured, online questionnaire. The collected data included demographics, mental health, physical health, eating habits, body weight, sleep, and nicotine intake. This study was conducted between September 25, 2020, and December 31, 2020.

**Results:**

Seventy respondents from Qatar participated in the study. Pre-pandemic healthy lifestyle behaviors (physical activity, healthy diet, sleep, etc.) showed no significant differences between people with and without disabilities. However, perceived changes in mental and physical health and eating habits during the pandemic lockdown differed between people with and without disabilities. People with disabilities reported worsening mental health (64.7%), physical health (53%), and dietary habits compared to the pre-pandemic levels. Furthermore, the more severe the disability, the more significant is its impact on mental and physical health.

**Conclusion:**

This study indicated that the pandemic has had a significant impact on the mental and physical health of people with and without disabilities in Qatar. These findings reveal how closely individuals with disabilities and their lifestyle choices are related to their mental and physical well-being. This will enable the development of specific interventions for similar situations in the future.

## 1 Introduction

COVID-19 is a disease caused by SARS Coronavirus. It is a contagious respiratory illness that triggers the immune system and causes acute damage to other organs. The sequelae of COVID-19 are associated with high morbidity and mortality rates. The World Health Organization (WHO) declared it a pandemic in March 2020. This pandemic has created an infodemic (1, 2). The global response initiated Public Health and Social Measures (PHSM) (3), including using personal protective measures following cough hygiene, physical distancing, avoiding gatherings, and international travel. The rapid spread of the illness has had a significant impact on both the healthcare sector and the global economy. The policies adopted to flatten the curve reduced transmission and prevented further overwhelming of the health care system. The WHO Coronavirus dashboard for Public Health and Social Measures Severity Index (PHSM) was reviewed, and there was no reported data for Qatar (4). In Qatar, the Ministry of Public Health (5) developed the COVID-19 National Response Plan. Partial curfews were enforced. Schools, colleges, and non-essential companies were closed, and international travel was suspended, compulsory Covid testing and temperature checks at all public spaces, and the use of Ehteraz application were enforced; healthcare workers used personal protective equipment.

People with disabilities commonly encounter an unequal and inaccessible world. “They were the hardest hit by the pandemic” said United Nations General Secretary Antonio Guterres. “COVID-19 laid bare the persistent barriers and inequalities faced by the world’s 1 billion persons with disabilities” he said (6). A few months prior to that the ‘Doha Declaration’ following the Doha International Conference on Disability and Development in December 2019 came up with recommendations to avoid fragmented services and reduce discrimination against individuals with disability (7). Qatar has been sensitive to the inclusion of persons with disabilities, as evidenced by the Qatar National Vision 2030 (8). Aspetar, the premier orthopedic and sports hospital, provided Qatar National Physical Activity Guidelines for all individuals in 2014 (9). Qatar also has a National dietary guideline called a shell-shaped plate that consists of six food groups: vegetables, fruits, cereals, starchy vegetables, legumes, milk, dairy products, and alternatives, and fish, poultry, meat, and alternatives. It is recommended to eat a variety of healthy food choices from these six food groups daily (10). In this backdrop COVID-19 pandemic began in early 2020.

Ammar et al. (11) from Germany did an electronic survey in June 2020 to study the effects of home confinement on lifestyle behaviors (ECLB) before and during the COVID-19 pandemic revealing that it altered physical activity and eating behaviors in a health-compromising direction. Public health and social measures were executed in varying time durations and intensities to prevent the spread and transmission of Covid 19 especially before vaccination. The effects of COVID-19 have been studied over a wide range of sociodemographic variables. However, there is a gap in the information and research related to the effects of COVID-19 on people with disabilities. A study from the United States explored the experiences of nurses who looked after individuals with intellectual disabilities, cerebral palsy, and autism spectrum disorder at the start of the pandemic. In April 2020, as part of a multiple method study, they used manifest content analysis to evaluate nurses to an online survey question about their experience of being a developmental disability nurse while encountering challenges to meeting the basic care needs of their clients. The nurses had lived with fear of COVID-19 and stress while helping others understand and cope, navigating a changing landscape, and feeling left out (12).

The World Health Organization started a campaign #HealthyAtHome (13), encouraging global citizens to stay physically active, take care of mental health, advocate healthy parenting, eat healthily, and quit tobacco during the COVID-19 pandemic lockdown. However, little information is available from the perspective of people with disabilities.

This study was initiated by a group of physiatrists who were members of an international taskforce. Physiatrists (physicians who are qualified in the field of physical medicine and rehabilitation) are referred to as quality of life physicians for persons with impairments and disabilities. This Task force of International Society of Physical Medicine and Rehabilitation (ISPRM) on Physical Activity in Persons with Disabilities (PAPD) decided that it was a crucial time to look at the impact of Covid 19 pandemic from the perspective of persons with disabilities. Hence, an international study in seven languages, using an online questionnaire, was initiated. The questionnaire used the ICF (International Classification of Functioning, Disability, and Health) framework of functioning and disability. Qatar and Morocco participated in the Middle East North Africa (MENA) region.

The primary global survey data of 3,550 responses by Tuakli-Wosornu et al. showed a 52% decrease in physical activity and a significant difference in the physical and mental health of those with and without disabilities (14). Azzouzi et al. found a negative impact of the COVID -19 pandemic on individuals with non-communicable diseases. A significant decline in physical health and a statistically insignificant decline in mental health have been reported (15). Another analysis from South Korea examined the impact of 189 respondents from their country compared with respondents around the world. They found no significant differences in the physical health and eating habits of individuals with and without disabilities in South Korea during the lockdown. However, they found that perceived mental health was worse during the lockdown for those with and without disabilities (16). There is a female preponderance in Mexico. This study showed that 53.3% of participants had decreased physical activity during the pandemic. Similarly, mental health is greatly affected. They found a disparity in the responses between those with lower education, obesity, and strict lockdown measures and those with no self-perceived impairments or income changes impacted by the COVID-19 pandemic (17). In Qatar, we aimed to study the respondents with and without disability. Lifestyle behaviors have impacted mental and physical health during the pandemic. Prior to the pandemic, Al-Thani et al. found that 83% of adults did not consume the recommended fruit and vegetable intake, as recommended by the guidelines, and 70% were overweight or obese (18). The primary objective of this study was to assess the initial impact of the COVID-19 pandemic on perceived mental well-being, physical activity levels, healthy eating habits, sleep habits, sedentariness, and nicotine intake in persons with disabilities in Qatar. The secondary objective was to compare the perceived effects of the COVID-19 pandemic on people with and without disabilities living in a community in Qatar.

## 2 Methods

### 2.1 Study design and participants

This was a cross-sectional web-based study to assess the initial impact of the COVID-19 pandemic on the perceived mental well-being, physical activity levels, healthy eating habits, sleep habits, sedentariness, and nicotine intake of people with disabilities in Qatar. The study participants were staff members at the Qatar Rehabilitation Institute and their family members. Individuals with all disabilities (mobility, intellectual, medical conditions, hearing impaired and visually impaired) living in the community also participated in this study. All participants were over 18 years of age and anonymous to participate in the study. The Institutional Review Board waived the requirement for informed consent. The study was conducted from September 25 to December 31, 2020, corresponding to the gradual removal of travel restrictions. Data were collected using an online questionnaire shared by members of the research team.

### 2.2 Outcome measures

The International Society of Physical Medicine and Rehabilitation (ISPRM) task force on physical activity in persons with disabilities (PAPD) developed an online multilingual questionnaire in Arabic, Chinese, English, French, Russian, Spanish, and Korean languages. Arabic and English questionnaires were used in this study. The online Qualtrics survey consisted of 24 questions with multiple answer options. The initial part of the survey collected demographic information, such as gender, age, nationality, educational level, and employment status. The next part of the survey used the Washington Group Questions (WGQ) (19) to collect information on health conditions and ICF *International Classification of Functioning, Disability and Health:* with components of body function and activity participation (20). The body function components used to assess the current level of functioning included in the questionnaire were (1) mental functions International Classification(intellect, attention, memory, learning, emotional regulation, and language) (2), voice and vocalization (3), circulatory systems (4), digestive, and (5) movement-related (joint mobility, muscle power tone, and strength) functions. The International Classification of Functioning (ICF) activities and participation components were (1) learning and applying knowledge (reading, writing, problem-solving, speaking with conversations) (2); mobility lifting/carrying/picking up/grasping/walking (3); self-care, washing, toilet activities, eating, and drinking (4); domestic life, shopping, cooking, cleaning house, washing dishes, and laundry (5); community, social, and civic life (recreation/religious); and (6) relationships with strangers, family, friends, and colleagues. Responses were rated as no functioning, some functioning, moderate functioning, or full functioning.

The healthy lifestyle behavior components inquired were physical activity for 30 min per day, diet (fruit and vegetables 5 servings/day), sleep 7–9 h, in days per week, uninterrupted hours of sitting/reclining, nicotine intake, and before and after the covid 19 lockdown restriction. Consumption of alcohol was not included as it is a taboo. Changes in physical wellness, mental and emotional wellness (anxiety, depression, sadness, and connectedness), and weight gain were also investigated, and no detailed physical and mental indicators were used as it was a holistic survey and did not focus on individual components. Mental health and wellness were inferred from the above components as pillars of lifestyle medicine for psychological well-being (21).

The COVID-19 pandemic lockdown enforced by the government also advised rules for lockdown (shelter in place), quarantine, wearing of masks, social distancing, and was associated with financial and job loss. Individuals chose the options which applied to them. The pre-and post-lockdown ailments and level of functioning of those with and without disabilities were recorded. The physical activity frequency of 30-minute exercise per week, fruit and vegetable (5 servings) intake per week, nicotine intake, sleep, duration of sedentariness, and weight gain were recorded. Sedentary time is the duration (hours/day) of uninterrupted time spent watching television or on mobile devices. Mental health and emotional wellness (i.e., anxiety, depression, sadness, and connectedness) were rated the same as before, better, or worse than before.

The survey was available on the ISPRM website (ISPRM.org) and on Facebook, Instagram, and Twitter. The PAPD Task Force members used their network of colleagues at work, friends, and family to recruit the participants. The survey used social media platforms, such as WhatsApp, Facebook, and email. The initial target was 1,000 respondents from multiple centers worldwide, and approximately 200 from Qatar. A total of 3,550 responded, of which only 70 were from Qatar.

In the Qatar Rehabilitation Institute, the survey was circulated among staff by email. The questionnaires were sent by email and WhatsApp to individuals with visual, hearing, and movement limitations, including the vison, hearing impaired, and Paralympic association of Qatar, to circulate the survey with their peers. Responses from individuals with autism, cerebral palsy, and intellectual impairment were provided by their parents. Respondents with disabilities self-declared that they had a disability in body function or participation in the online questionnaire. Since it was a virtual survey, we accepted the responses to their assessments.

### 2.3 Statistical analysis

Descriptive and inferential statistics were used to analyze the data. Categorical variables were presented as numbers and percentages. The *χ*^2^ test was used to compare the differences between groups with and without disabilities. All statistical tests were performed using the Statistical Package for the Social Sciences (SPSS) version 28 (IBM Corp., Armonk, NY, United States). The significance level in the present study was set at *p* < 0.05, which represents the statistical significance threshold. This analytical method was intended to investigate the demographic features and lifestyle aspects of respondents, distinguishing between those with and without disabilities. Statistical comparisons were designed to provide light on variations in mental well-being and physical activity and the influence of the COVID-19 pandemic on both groups.

## 3 Results

### 3.1 Demographic data

Seventy people from Qatar responded to the questionnaire, with a response rate of 23%. The responses were complete and did not contain any unanswered questions.

The female sex predominance rate was 53%. The remaining 47% of patients were men. In this study, 46% of persons were less than 40 years old, and 54% were more than 40 years. The educational level of the respondents was as follows:63% were postgraduates and the others had completed school or graduate studies. 95.7% were employed full- or part-time, whereas the rest were homemakers or unemployed. The results showed that 24.5% of respondents without disabilities had (non-communicable diseases) such as diabetes, hypertension, chronic respiratory disease, obesity, or heart disease, compared to 47% of persons with disabilities. 24% were disabled (Table 1).

**Table 1:** Socio-demographic details.

	Number	Percentage
Gender		
Male	33	47.1
Female	37	52.9
Age group		
<40	32	45.7
>40	38	54.3
Education		
Postgraduate	44	62.9
Graduate or less	26	37.1
Employment status		
Employed	67	95.7
Not employed	3	4.3
Comorbidities	21	30.0
Disability	17	24

All individuals without disabilities in body function, activities, or participation were fully functioning (100%). However, among individuals with disabilities these components were fully functional in terms of mental capacity (82.4%), visual/hearing (93.3%), vocal (86.7%), circulatory (84.6%), digestive (71.4%), movement (80%), communication (94%), mobility (75%), self-care (87.5%), domestic life (53%), social (43.8%), and relationships (50%).

### 3.2 Lifestyle before the COVID-19 pandemic

Physical exercise for 30 min/day is recommended by the WHO Health Organization. Of those without disabilities, 86.8% exercised, while 75% of those with disabilities reported that they exercised a few days per week. Persons without and with disability who exercised 2–4 days per week at 65 and 50%, respectively (Table 2).

**Table 2:** Disability-wise comparison factors before COVID.

	Without disability (53)	With disability (17)	Value of *p*
Gender			
Male	22 (41.5%)	11 (64.7%)	0.09
Female	31 (58.5%)	6 (35.3%)	
Age			
<40	19 (35.8%)	13 (76.5%)	0.003*
>40	34 (64.2%)	4 (23.5%)	
Education			
Post-Graduate	36 (67.9%)	8 (47.1%)	0.121
Graduate or less	17 (32.1%)	9 (52.9%)	
Employment status			
Employed	51 (96.2%)	16 (94.1%)	0.709
Not employed	2 (3.8%)	1 (5.9%)	
Comorbidities			
No	40 (75.5%)	9 (52.9%)	0.70
Yes	13 (24.5%)	8 (47.1%)	
***ICF Body Function Component***			
Mental (intellect, attention, memory, emotional regulation)			
Full functioning	53 (100.0%)	14 (82.4%)	0.008*
Moderate functioning	0 (0.0%)	2 (11.8%)	
Some functioning	0 (0.0%)	1 (5.9%)	
Seeing/hearing/balance			
Full functioning	53 (100.0%)	14 (93.3%)	0.05
Moderate functioning	0 (0.0%)	1 (6.7%)	
Voice/vocalization			
Full functioning	53 (100.0%)	13 (86.7%)	0.007*
Moderate functioning	0 (0.0%)	2 (13.3%)	
Circulatory			
Full functioning	53 (100.0%)	11 (84.6%)	0.01*
Moderate functioning	0 (0.0%)	1 (7.7%)	
No functioning	0 (0.0%)	1 (7.7%)	
Digestion			
Full functioning	53 (100.0%)	10 (71.4%)	0.001*
Moderate functioning	0 (0.0%)	3 (21.4%)	
Some functioning	0 (0.0%)	1 (7.1%)	
Movement (joint mobility, muscle power tone, strength)			
Full functioning	53 (100.0%)	12 (80.0%)	0.004*
Moderate functioning	0 (0.0%)	2 (13.3%)	
No functioning	0 (0.0%)	1 (6.7%)	
***ICF Activities & Participation component***			
Learning knowledge and communication (reading, writing, problem solving)			
Full functioning	53 (100.0%)	16 (94.1%)	0.07*
No functioning	0 (0.0%)	1 (5.9%)	
Mobility (lifting and carrying, walking)			
Full functioning	53 (100.0%)	12 (75.0%)	0.001*
Moderate functioning	0 (0.0%)	2 (12.5%)	
No functioning	0 (0.0%)	2 (12.5%)	
Self-care			
Full functioning	53 (100.0%)	14 (87.5%)	0.03*
Some functioning	0 (0.0%)	1 (6.3%)	
No functioning	0 (0.0%)	1 (6.3%)	
Domestic life			
Full functioning	53 (100.0%)	8 (53.3%)	0.001*
Moderate functioning	0 (0.0%)	4 (26.7%)	
Some functioning	0 (0.0%)	1 (6.7%)	
No functioning	0 (0.0%)	2 (13.3%)	
Social life (recreation, religion)			
Full functioning	53 (100.0%)	7 (43.8%)	0.001*
Moderate functioning	0 (0.0%)	4 (25.0%)	
Some functioning	0 (0.0%)	4 (25.0%)	
No functioning	0 (0.0%)	1 (6.3%)	
Relationships (strangers, family, friends)			
Full functioning	53 (100.0%)	8 (50.0%)	0.001*
Moderate functioning	0 (0.0%)	3 (18.8%)	
Some functioning	0 (0.0%)	4 (25.0%)	
No functioning	0 (0.0%)	1 (6.3%)	
Physical exercise			
***Healthy lifestyle Behaviors – Pre pandemic***			
Have exercised	46 (86.8%)	12 (75.0%)	0.259
Do not exercise	7 (13.2%)	4 (25.0%)	
Frequency of 30 minutes exercise (Days/week)			
More than 4 days a week	9 (19.6%)	3 (25.0%)	0.601
2–4 days per week	30 (65.2%)	6 (50.0%)	
0–1 day per week	7 (15.2%)	3 (25.0%)	
Fruit and vegetable intake			
Had fruit and veg	50 (94.3%)	13 (76.5%)	0.03*
No fruit and veg	3 (5.7%)	4 (23.5%)	
Frequency of Fruit and veg intake			
More than 4 days a week	15 (30.0%)	3 (23.1%)	0.8
2–4 days per week	30 (60.0%)	8 (61.5%)	
0–1 day per week	5 (10.0%)	2 (15.4%)	
Pre- pandemic sleep			
Sleep affected	47 (88.7%)	15 (88.2%)	0.96
Sleep not affected	6 (11.3%)	2 (11.8%)	
Duration of sleep 7–9 h per day			
More than 4 days a week	24 (51.1%)	3 (20.0%)	0.1
2–4 days per week	14 (29.8%)	8 (53.3%)	
0–1 day per week	9 (19.1%)	4 (26.7%)	
Sedentariness			
Not sedentary	52 (98.1%)	15 (88.2%)	0.80
Sedentary	1 (1.9%)	2 (11.8%)	
Duration of pre-sedentariness			
<2 h/day	7 (13.5%)	2 (13.3%)	0.9
2–4 h/day	27 (51.9%)	8 (53.3%)	
>4 h/day	18 (34.6%)	5 (33.3%)	
Nicotine consumption			
Smoking	4 (7.7%)	2 (11.8%)	0.6
Nonsmoking	48 (92.3%)	15 (88.2%)	

*Statistically significant (*p* < 0.05).

23% with disabilities responded that they ate at least five servings of fruit and vegetables four times a week, whereas 30% of those without disabilities ate five servings.

20% of those with disabilities responded that they slept for more than 7 h, 4 days a week, while 51% without disabilities slept for the same frequency and duration per week.

More than half of the respondents reported being sedentary for 2–4 h/day. People with disabilities (11.8%) consumed more nicotine products than those without disabilities (7.7%).

### 3.3 Lifestyle during the COVID-19 pandemic

In Qatar, our results showed that people with disabilities had more mask use by the public (94.1%). Among those with disabilities, 47.1% were quarantined. The non-disabled group followed social distancing (88.5%) and experienced lockdown (53.8%). In contrast, comparing patterns in terms of the effects on economic impact, people with disabilities reported a considerably higher percentage of income loss or reduction (29.4%) than their non-disabled counterparts (9.6%, *p* = 0.04*). Their lifestyles changed, as revealed by 23.5% of participants experiencing changes in physical exercise and 47% experiencing changes in sleep duration. However, 46% of those without disabilities and 40% of those with disabilities spent 2-4 hrs in sedentary activities, such as watching television (Table 3).

**Table 3:** Disability-wise comparison factors during COVID.

	Without disability	With disability	Value of *p*
Experienced lockdown			
Yes	28 (53.8%)	8 (47.1%)	0.627
No	24 (46.2%)	9 (52.9%)	
Wore mask in public			
Yes	46 (88.5%)	16 (94.1%)	0.503
No	6 (11.5%)	1 (5.9%)	
Maintained social distancing			
Yes	46 (88.5%)	15 (88.2%)	0.980
No	6 (11.5%)	2 (11.8%)	
Income loss/Income reduction			
Yes	5 (9.6%)	5 (29.4%)	0.04*
No	47 (90.4%)	12 (70.6%)	
Total Job loss/Unemployment			
Yes	1 (1.9%)	2 (11.8%)	0.08
No	51 (98.1%)	15 (88.2%)	
Quarantined			
Yes	8 (15.4%)	8 (47.1%)	0.007*
No	44 (84.6%)	9 (52.9%)	
Physical exercise change			
The same as before	14 (26.9%)	4 (23.5%)	0.43
More than before	8 (15.4%)	5 (29.4%)	
Less than before	30 (57.7%)	8 (47.1%)	
Fruit intake change			
The same as before	27 (51.9%)	6 (35.3%)	0.13
More than before	18 (34.6%)	5 (29.4%)	
Less than before	7 (13.5%)	6 (35.3%)	
Sleep change			
The same as before	36 (67.9%)	8 (47.1%)	0.15
More than before	5 (9.4%)	1 (5.9%)	
Less than before	12 (22.6%)	8 (47.1%)	
Nicotine consumption change			
The same as before	4 (57.1%)	2 (40.0%)	0.59
More than before	2(28.6%)	1 (20.0%)	
Less than before	1 (14.3%)	2 (40.0%)	
Sedentariness (Screen time)			
< 2 h/day	17 (32.7%)	4 (26.7%)	0.62
2–4 h/day	24 (46.2%)	6 (40.0%)	
>4 h/day	11 (21.2%)	5 (33.3%)	
Perceived physical health change			
The same as before	22 (41.5%)	4 (23.5%)	0.38
Better than before	8 (15.1%)	4 (23.5%)	
Worse than before	23 (43.4%)	9 (52.9%)	
Perceived mental health change			
The same as before	16 (30.2%)	5 (29.4%)	0.89
Better than before	5 (9.4%)	1 (5.9%)	
Worse than before	32 (60.4%)	11 (64.7%)	
Perceived eating habit change			
The same as before	25 (47.2%)	6 (35.3%)	0.68
Better than before	12 (22.6%)	5 (29.4%)	
Worse than before	16 (30.2%)	6 (35.3%)	
Weight gain			
Yes	27 (51.9%)	8 (47.1%)	0.72
No	25 (48.1%)	9 (52.9%)	
Weight loss			
Yes	8 (15.7%)	5 (29.4%)	0.221
No	43 (84.3%)	12 (70.6%)	
Weight gain lbs			
< 2.5 kg (5lbs)	12 (44.4%)	4 (50.0%)	0.72
2.6-5 kg (5-11lbs)	13 (48.1%)	3 (37.5%)	
5.4–9.5 kg (12-21lbs)	1 (3.7%)	1 (12.5%)	
10 kg or more (22lbs or more)	1 (3.7%)	0 (0.0%)	

*Statistically significant (*p* < 0.05).

The perceived mental health change in respondents with a disability during the first COVID-19 lockdown was worse than before (64.7%). Perceived physical health change was also worse than before (52.9%). Worsening of eating habits was observed in 35.3% of participants. Those who experienced weight loss and gain were 29.4 and 47%, respectively, compared with those without disability. However, individuals without disabilities experienced marginally more weight gain (51.9%) and increased nicotine use (28.6%) than those with disabilities.

## 4 Discussion

This survey is unique because it is the only study in Qatar that captures the impact of the COVID-19 lockdown on the lifestyle and perceived mental and physical health of persons with disabilities and compares it with those without disabilities. This study is part of an international study that collected data from 65 countries. Each country has responded to the COVID-19 pandemic based on its unique circumstances, resources, and government strategies.

The World Health Organization started a campaign #HealthyAtHome (13), encouraging global citizens to stay physically active, take care of mental health, advocate healthy parenting, eat healthily, and quit tobacco during the COVID-19 pandemic lockdown. The campaign highlighted the adverse effects of the excessive consumption of newsfeeds, social media, and screen time.

In Qatar, the self-perceived worsened self-perceived mental health and emotional wellness changes pandemic were 60.4% in individuals without disabilities and 64.7% in those with disabilities. This could be attributed to the widening of pre-existing gaps in access to wellness programs, mobility, and physical activity in people with disabilities (22). As mentioned earlier Al-Thani et al. there are inherent gaps and substantial shortfall in the lifestyle of individuals living in Qatar. He had found that 83% of adults did not have a balanced diet in consuming the recommended fruit and vegetable intake, and 70% were overweight or obese (18).

This was similar to the primary study by Tuakli-Wosornu et al. (14), which observed that 61.37% of participants were not disabled, 69.58% had mild to moderate disability, and 75% had severe disability and reported deterioration in their mental health. Another report from South Korea by Tuakli-Wosornu et al. revealed that 65% of South Korean respondents with and without disabilities had worsening mental status. Stratton et al. reported that Mexican women had a 73.8% worsening of mental health at that time, and this finding was due to isolation, lack of social life, boredom, or mortality in the family and community (17). Salanti et al. reported a relationship between the COVID-19 pandemic and mental health in their meta-analysis (23). They found an increased prevalence of depression and anxiety during the pandemic. They proposed that there may be variation in how the population responds to the psychological stress generated by the pandemic and its control measures. Mittal et al. (24) described the COVID-19 Pandemic as a lifestyle disorder that spared nobody. She observed a fear of infection, lockdown, social isolation, anger, and violence against women, children, and immigrants. She urged the requirement of hotlines, shelters, crisis centers, counseling, and revamping the healthcare system.

Self-perceived physical health change in Qatar worsened in 43.4% of individuals without disabilities and 52% of those with disabilities. Tuakli-Wosornu et al. primary study (14) reported that 40% of the participants were not disabled, 48.3% had mild to moderate disability, and 56% had severe disability and reported deterioration in their physical health. In our study, 58% of individuals without disabilities and 47% of persons with disabilities exercised 2–4 days/week, it is slightly better than that in the primary study where 41% (with and without disabilities) exercised at the same frequency. This could be due to the decision of the Ministry of Public Health (MOPH), which kept public parks and niches open for walking and running throughout the pandemic. However, in Qatar, the duration of sedentary activity for 2–4 h was 46% (without disability) and 40% (with disability), while the primary study revealed 45% in persons with and without disability. These data should be viewed in the background that 30% of men and 44% of women in Qatar do not usually do any physical activity, as indicated by the International Physical Activity Questionnaire (IPAQ) questionnaire in the Qatar Biobank report (25). A rapid review by Park et al. reported a decrease physical activity during the COVID-19 pandemic. They suggested that it is detrimental to physical and mental health (26).

Our study found a marginal worsening in eating habits in servings of recommended fruit and vegetable intake in persons with and without disabilities at 35 and 30%, respectively, while the primary study reported 27 and 35%, respectively. Ben Hassen et al. did a study on impact of food behavior and consumption in Qatar during the COVID-19 pandemic and found their respondents shifted to healthier diets, used domestic produce consumed more home cooked food, used more online grocery shopping and avoided panic buying. They found that 32.4% consumed more fruits and vegetables and 28.7% ate less candy, cookies, and pastries. However, this study did not quantify the servings consumed per day (27). There was a significant decrease in time spent in physical activity and an increase in sitting/reclining time. Abed Alah et al. in their study of lifestyle impact in Qatar, found that 27.8 and 33.2% perceived that their overall diet had become less healthy or healthier, respectively. This study used statements such as the tendency to eat fatty food, junk food, sugar/chocolate, vegetables, and fresh fruit food as outcome measures (28).

Weight gain in our study participants was slightly higher in those with disability (52%) than in those without disability (47%). A primary study by Ben Hassen et al. found weight gains of 42 and 48% in those with and without disability, respectively (27). Abed Alah et al. from Qatar found more than half the participants in their study 52.2% self-reported weight during the stay at home. They observed a weight gain of 3–6 kgs. They inferred that this was due to a reduction in exercise duration and an increase in sedentary behavior (28). The current prevalence of obesity and overweight in Qatar is 41.5 and 35.5%, respectively, as reported by the Qatar Biobank (29).

In our study, nicotine consumption increased by 28.6 and 20%, respectively, and 57 and 40% remained the same in those without and with disability, respectively. Tuakli-Wosornu et al. reported that 91 and 90% of them did not change their habits and continued the same as before. In Qatar, shisha smoking is more common than cigarette smoking is. Shisha is a heated tobacco usually flavored with fruit or molasses sugar and is a common cultural leisure activity. The Qatar Biobank report stated that 21% of men and women smoked cigarettes, while 33% smoked shisha (29).

Sleep changes were the same in 68% of persons with disabilities and 47% of those without disability. Jahrami et al. in their systematic meta-analysis reported an association between COVID-19 and sleep (30). They found 41.6% sleep disturbance in persons with special needs, which is less affected than those with Covid 19 and children and adolescents, but more affected than the general population. They suggested that sleep problems associated with pandemics were due to improper wake-up and sleep time due to poor physical and mental health. O’Sullivan et al. reported an association between COVID-19, social isolation, and loneliness in a multi-country study (31). They observed that COVID-19 has increased social isolation, loneliness, and poor mental health. It is evident from these studies that maintaining proper lifestyle practices, adequate sleep, regular exercise, and a balanced diet are protective factors for mental and physical health during the pandemic. Kim et.al found changes in sleep habits were associated with depression due to restrictions in outside activities, social distancing, and increased family conflicts (32).

Over the past three decades, Qatar has experienced phenomenal economic growth. Rapid urbanization and mechanization have led to accelerated lifestyle changes, with an increased burden of non-communicable diseases causing mortality in 69% of the cases (33). Earlier, the WHO STEP surveillance study in 2012 revealed an incidence of 70% overweight and 41% obesity, 71% did not perform any vigorous physical activity, and 90% did not eat five servings of fruit and vegetables daily (34).

Our survey confirmed that the COVID-19 lockdown in Qatar affected everyone, but individuals with disabilities were disproportionately affected, as in other parts of the world. Limited access to information and healthcare, public spaces, and a sense of isolation must be structured when planning for persons with disabilities. Awareness of intrapersonal, interpersonal, and structural constraints (35) faced by people with disabilities can help Qatar prepare for future pandemics and disasters.

## 5 Strengths and limitations

This study’s strength allows a thorough understanding of the impact of the COVID-19 lockdown in Qatar on persons with disabilities. Second, it combines data on mental and physical well-being and provides comprehensive information on an individual’s well-being.

The limitations of this study include the recall bias of the self-reported questionnaire, hospital-based study population, small sample size, and low response rate. There could have been reluctance to share personal information about their disabilities online. Understanding these questions and the digital barriers may be another reason. Participation in housework may not have been quantified as physical activity. This survey required access to an Internet connection and technology such as smartphones and computers. This study was cross-sectional and did not examine trends over time.

In conclusion, the COVID-19 pandemic’s first lockdown negatively affected mental well-being by 65% and physical activity by 53% of people with and without disabilities in Qatar. About a quarter (24%) of those with disabilities experienced changes in physical exercise, 47% experienced changes in sleep, more than one-third (35%) experienced changes in dietary habits, and 47% experienced weight gain. Lifestyle changes were enhanced because of probable disability discrimination and fear of infection. These findings show how closely people with disabilities and their lifestyle choices relate to their mental and physical well-being. These observations will be beneficial in developing specific strategies for future crises.

## Data availability statement

The original contributions presented in the study are included in the article, further inquiries can be directed to the corresponding author.

## Ethics statement

Studies involving human participants were reviewed and approved by the Human Research Protection Program Institutional Review Board of Yale University and Medical Research Center, the Institutional Review Board of Hamad Medical Corporation. The Institutional Review Board waived the requirement for informed consent.

## Author contributions

UP: Conceptualization, Data curation, Funding acquisition, Investigation, Methodology, Project administration, Resources, Supervision, Validation, Writing – original draft. BS: Formal analysis, Validation, Visualization, Writing – review & editing. IK: Formal analysis, Validation, Visualization, Writing – review & editing. RS: Investigation, Validation. FA: Investigation, Validation. PA: Investigation, Validation. SH: Investigation, Validation. LZ: Investigation, Validation.
